# Central Obesity and Estimated Coronary Heart Disease Risk Among Jordanian Women Aged 30–50 Years: A Framingham Risk Score Analysis

**DOI:** 10.1155/jnme/3899686

**Published:** 2026-11-14

**Authors:** Omar Khaled Alboqai, Bayan Obeidat, Mohammad A. Ebqa’ai, Mohammed O. Ibrahim, Nermeen Bleedy, Tamara Ibrahim Shafiq Al-Sa’adi, Baha M. Abu Salma, Dima Alkadri, Nizar Alrabadi, Mohammed Gamal

**Affiliations:** ^1^ Department of Food Science and Nutrition Faculty of Agriculture Jerash University Jerash Jordan jpu.edu.jo; ^2^ Department of Nutrition and Food Technology Faculty of Agriculture Jordan University of Science and Technology Irbid Jordan just.edu.jo; ^3^ Department of Chemistry Faculty of Science Yarmouk University Irbid 21163 Jordan yu.edu.jo; ^4^ Department of Nutrition and Food Technology Faculty of Agriculture Mutah University Karak Jordan mutah.edu.jo; ^5^ Physical Therapy Department Faculty of Applied Medical Science Jerash University Jerash Jordan jpu.edu.jo; ^6^ Food Science and Nutrition Nutritionist at Al-Sarih Medical Consulting Center Irbid Jordan

**Keywords:** coronary heart disease, Framingham risk score, Jordan, obesity, physical activity, waist-to-height ratio, women’s health

## Abstract

**Background:**

Obesity is a well‐established modifiable risk factor for cardiovascular disease (CVD), particularly coronary heart disease (CHD). Its high prevalence among Jordanian women aged 30–50 years highlights the need for early cardiovascular risk assessment to support targeted preventive strategies.

**Objective:**

To estimate the 10‐year risk of CHD among Jordanian women aged 30–50 years using the Framingham risk score (FRS) and to examine the associations between general obesity, central obesity, cardiometabolic risk factors, and estimated CHD risk.

**Methods:**

A cross‐sectional study was conducted among 230 Jordanian women aged 30–50 years who were recruited using a multistage cluster sampling technique. Standardized anthropometric measurements, including body weight, height, and waist circumference (WC), were obtained to calculate body mass index (BMI) and waist‐to‐height ratio (WHtR). Blood pressure was measured according to standardized procedures, and fasting blood samples were analyzed to determine fasting blood glucose and lipid profile parameters. Smoking status and physical activity were assessed using structured questionnaires. The 10‐year risk of CHD was estimated using the FRS. Descriptive and inferential statistical analyses were performed to evaluate the associations between obesity indices and estimated CHD risk.

**Results:**

General obesity was identified in 39.1% of participants, while 40.9% had central obesity based on WC (≥ 88 cm). Nearly half of the participants (47.0%) were current smokers, and 21.3% reported low levels of physical activity. According to the FRS, 66.1% of participants were classified as having low CHD risk, 16.5% as moderate risk, and 17.4% as high risk. Higher BMI and central obesity were significantly associated with elevated blood pressure, fasting blood glucose, triglyceride levels, and LDL cholesterol, as well as lower HDL cholesterol (all *p* < 0.001). Smoking, physical inactivity, and both general and central obesity were significantly associated with an increased estimated risk of CHD.

**Conclusion:**

Jordanian women aged 30–50 years exhibited a substantial burden of both general and central obesity, accompanied by an increased estimated risk of CHD. These findings underscore the importance of routine cardiovascular risk assessment and comprehensive lifestyle interventions focusing on weight management, smoking cessation, and regular physical activity. Because the FRS has not been specifically validated in Jordanian women, the estimated absolute cardiovascular risk should be interpreted with caution.

## 1. Introduction

Women aged 30–50 years undergo important hormonal and physiological changes, and many experience the menopausal transition during this period. These changes, particularly the gradual decline in estrogen levels, are associated with increased visceral fat accumulation, insulin resistance, dyslipidemia, and elevated cardiovascular risk, thereby increasing susceptibility to cardiometabolic disorders, including coronary heart disease (CHD) [1–5]. However, the timing and progression of the menopausal transition vary considerably among women. As obesity becomes increasingly prevalent during midlife, understanding its contribution to cardiovascular risk is essential for developing effective preventive strategies.

Obesity is a chronic, multifactorial disease and one of the leading contributors to the global burden of noncommunicable diseases. It is strongly associated with hypertension, type 2 diabetes mellitus, dyslipidemia, metabolic syndrome, and cardiovascular disease (CVD). Excess adipose tissue promotes chronic low‐grade inflammation, oxidative stress, endothelial dysfunction, and insulin resistance, all of which accelerate atherosclerosis and increase the risk of CHD [1–5].

This public health challenge is particularly evident in Jordan, where obesity among women has reached alarming levels. National surveys indicate that approximately 29.3% of women aged 15–49 years are overweight and 30.2% are obese [6]. Other studies have reported that nearly two‐thirds of Jordanian women are either overweight or obese [7], while metabolic syndrome affects approximately one‐third to one‐half of the adult population, with central obesity representing its most prevalent component [1, 6–10]. These findings highlight the urgent need for effective strategies to prevent obesity‐related cardiometabolic diseases among Jordanian women.

Central obesity has emerged as a stronger predictor of CVD than general obesity because it more accurately reflects visceral fat accumulation. Increased visceral adiposity contributes to chronic inflammation, oxidative stress, endothelial dysfunction, and activation of the renin–angiotensin–aldosterone system (RAAS), thereby promoting atherosclerosis and increasing cardiovascular risk [8, 10, 11]. Lifestyle‐related factors, particularly cigarette smoking and physical inactivity, further exacerbate these metabolic abnormalities and substantially increase the risk of CVD [8, 9]. CVD accounts for more than 40% of all deaths in Jordan, underscoring the urgent need for early identification and management of modifiable cardiovascular risk factors among women [7].

Despite the high prevalence of obesity and metabolic syndrome in Jordan, few studies have comprehensively evaluated the estimated 10‐year risk of CHD among Jordanian women aged 30–50 years using standardized cardiovascular risk assessment tools. Most previous studies have focused on individual cardiometabolic risk factors rather than integrating these variables into a validated risk prediction model. Furthermore, although the Framingham risk score (FRS) is widely used internationally, its performance has not been specifically validated in Jordanian women. Therefore, its estimated absolute cardiovascular risk should be interpreted with appropriate caution.

Accordingly, the present study aimed to estimate the 10‐year risk of CHD among Jordanian women aged 30–50 years using the FRS and to examine the associations between general obesity, central obesity, cardiometabolic risk factors, and estimated CHD risk.

## 2. Materials and Methods

### 2.1. Study Design and Setting

A cross‐sectional study was conducted between May 2025 and October 2025 in the Al‐Sarih area of Irbid Governorate, northern Jordan. This location was chosen for its characteristics as a semiurban community with a relatively homogeneous lifestyle, socioeconomic, and cultural background. The study adhered to the principles of the Declaration of Helsinki and received ethical approval from the Institutional Review Board of Jerash University (No. O/1/2025/2026).

### 2.2. Sample Size and Sampling Technique

A sample size was determined to ensure adequate estimates and appropriate distribution across body mass index (BMI) categories (normal weight, overweight, and obese) to meet the study’s objectives. A multistage cluster sampling technique was employed. The study area was first divided into five primary clusters. Within these clusters, households were selected systematically (every 30th house). From each selected household, one eligible woman was invited to participate. If more than one eligible woman from different BMI categories was present in a household, one participant was randomly recruited. If a household declined participation or did not meet the study criteria, the next household in the sequence was approached.

### 2.3. Participants

Participants were eligible for the study if they were married Jordanian women aged 30–50 years, not pregnant or breastfeeding, and were apparently healthy with no acute or metabolic illness within the two months prior to the study. Key exclusion criteria were then applied to ensure a homogenous sample: Individuals were excluded if they had any physical disabilities, were underweight (BMI < 18.5 kg/m^2^), or had a prior diagnosis of a serious chronic illness, including overt diabetes, hypertension, or established CVD. Furthermore, participants were excluded for taking medications that could affect energy metabolism or body composition, engaging in strenuous physical activity, or having experienced weight fluctuations of more than 5 kg in the 2 months preceding testing. Because detailed reproductive history, menstrual cycle characteristics, and hormonal assessments were not collected, participants were not clinically classified according to standardized menopausal staging criteria (e.g., STRAW+10). Consequently, eligibility was determined solely by age. 230 participants completed the full study procedures (80 with a BMI of 18.5–< 25 kg/m^2^, 60 with a BMI of ≥ 25–< 30 kg/m^2^, and 90 with a BMI ≥ 30 kg/m^2^). A total of 10 subjects were excluded based on the study criteria, as they had overt diabetes (*n* = 4), CVD (*n* = 3), and hypertension (*n* = 3). However, an elevated blood pressure value measured during the study was not itself an exclusion criterion and was retained for analysis. The remaining 230 individuals were included in the statistical analysis.

### 2.4. Assessment Procedures

Data were collected through structured interviews, questionnaires, anthropometric measurements, and biochemical analyses. Written informed consent was obtained from all participants prior to data collection, and all information was coded and handled with strict confidentiality.

1. Questionnaire assessment: A comprehensive, structured questionnaire was administered by trained interviewers to gather data on demographic, economic, and sociocultural characteristics, as well as smoking habits and medical history.
 Physical activity was assessed using an adapted instrument based on physical activity items described by Al‐Hazzaa for healthcare workers in the Gulf Cooperation Council countries [12]. The instrument was adapted to the objectives of the present study by combining some items, separating some items into distinct components, and excluding items primarily related to flexibility. The final adapted instrument comprised seven questions, with four scored components/items within each question. Questions 1, 2, 3, and 6 were each scored from 0 to 3 points; Questions 4 and 5 were each scored from 0 to 6 points; and Question 7 was scored from 0 to 4 points. The scores were summed to obtain a total physical activity score ranging from 0 to 28 points, with higher scores indicating higher physical activity.
 For the present study, the total score was operationally categorized into three score intervals: inactive (0–8 points), moderately active (9–17 points), and active (18–28 points). These cutoff points were established for the present study based on the scoring distribution of the adapted instrument and were not attributed to the original Al‐Hazzaa instrument [12].
 The internal consistency of the adapted instrument was assessed in the current study using Cronbach’s alpha, which was 0.86, indicating good internal consistency. However, the adapted instrument was not subjected to formal criterion‐related or construct‐validity testing in the present study. Therefore, the physical‐activity categories should be interpreted as operational classifications specific to this study rather than as validated clinical or population‐level thresholds, and findings related to physical activity should be interpreted cautiously.
2. Anthropometric and blood pressure measurements: All anthropometric measurements were performed by a team of two trained female nutritionists using standardized techniques and equipment [13]. Participants were measured barefoot and in light clothing, and each measurement was taken twice and averaged.
• Height: Measured to the nearest 0.1 cm using a portable stadiometer.
• Weight: Measured to the nearest 0.1 kg using a calibrated electronic scale (Seca, Germany).
• Waist circumference (WC): Measured to the nearest 0.1 cm at the narrowest point between the lowest rib and the iliac crest at the end of normal expiration.
• Hip circumference: Measured to the nearest 0.1 cm at the widest part of the buttocks; however, it was not included in further analysis.
• Blood pressure: Blood pressure was measured twice in the left arm of seated participants after a 15‐min rest using a calibrated electronic sphygmomanometer, and the average value was recorded.
3. Biochemical analyses after an overnight fast of 12–16 h, three venous blood samples were drawn by a medical laboratory technician. Samples for glucose analysis were collected in tubes containing sodium fluoride and potassium oxalate. Other samples were centrifuged at the Al‐Sarih Medical Consulting Center and transported in iceboxes to a private medical laboratory for analysis. Biochemical parameters, including triglycerides (TG), total cholesterol (TC), high‐density lipoprotein cholesterol (HDL‐C), low‐density lipoprotein cholesterol (LDL‐C), and fasting blood glucose (FBG), were measured using a Roche Cobas c311 analyzer (Roche Diagnostics, Switzerland, 2013).
4. Definitions and risk calculations
• Obesity indices: BMI was calculated as weight (kg) divided by height (m^2^). General obesity was classified as normal weight (18.5–24.9 kg/m^2^), overweight (25–< 30 kg/m^2^), and obesity (≥ 30 kg/m^2^). WC was categorized as < 80 cm, 80–< 88 cm, and ≥ 88 cm. The 80‐cm threshold was based on the International Diabetes Federation criterion for women, whereas the ≥ 88‐cm threshold was based on the National Cholesterol Education Program Adult Treatment Panel III (ATP III) criterion. Waist‐to‐height ratio (WHtR) was calculated as WC (cm) divided by height (cm) and categorized as < 0.50, 0.50–< 0.55, and ≥ 0.55 [14–16].
• Biochemical and BP cutoffs: Abnormal lipid levels were defined according to NCEP‐ATP III guidelines: TC ≥ 200 mg/dL, TG ≥ 150 mg/dL, LDL‐C ≥ 100 mg/dL, and HDL‐C < 50 mg/dL [14]. Elevated FBG was defined as ≥ 100 mg/dL [17]. Elevated blood pressure was defined as systolic BP ≥ 120 mmHg or diastolic BP ≥ 80 mmHg [18].

### 2.5. Estimation of 10‐Year CHD Risk

The estimated 10‐year risk of hard CHD was calculated using the sex‐specific, point‐based Framingham CHD risk algorithm based on risk‐factor categories developed by Wilson et al. [19] and adopted for clinical risk assessment by the National Cholesterol Education Program ATP III [20]. The analysis specifically used the female‐specific ATP III Framingham point system for estimation of 10‐year hard CHD risk. Hard CHD was defined as nonfatal myocardial infarction or coronary death.

The risk score was calculated from the following variables: age, TC, HDL‐C, systolic blood pressure (SBP), and current smoking status. Age‐ and cholesterol‐specific points were assigned according to the female‐specific ATP III tables. Because participants receiving antihypertensive treatment were excluded during participant selection, the untreated SBP point categories were used for all participants. The total point score was then converted to the corresponding estimated 10‐year hard CHD risk percentage using the female ATP III risk table [19, 20].

Estimated 10‐year hard CHD risk was categorized as low (< 10%), moderate (10–< 20%), or high (≥ 20%). Because the ATP III Framingham model was not specifically developed or validated for Jordanian women, the calculated values were interpreted as estimated risk rather than population‐specific absolute CHD risk.

### 2.6. Statistical Analysis

All data were entered and analyzed using IBM SPSS Statistics Version 28 (IBM Corp., Armonk, NY, USA). Data were checked for completeness, range errors, internal consistency, and missing values before analysis. The normality of continuous variables was assessed using the Shapiro–Wilk test together with visual inspection of histograms and Q–Q plots. Normally distributed continuous variables were expressed as mean ± standard deviation (SD), whereas categorical variables were presented as frequencies and percentages [*n* (%)].

Continuous variables were compared across BMI categories using one‐way analysis of variance (ANOVA), followed by Scheffé post hoc comparisons when the overall ANOVA was statistically significant. Associations between categorical variables were assessed using the chi‐square test.

Prevalence ratios (PRs) were estimated as crude, unadjusted measures of relative prevalence. For each cardiometabolic abnormality, PR1 was calculated as the observed prevalence among overweight participants divided by the observed prevalence among participants with normal BMI, whereas PR2 was calculated as the observed prevalence among obese participants divided by the observed prevalence among participants with normal BMI. The normal‐BMI group was used as the reference category (PR = 1.00). Thus, no covariate adjustment or multivariable regression model was used for the PR estimates, and consequently no model‐fit assessment was applicable to these manually calculated crude PRs.

The corresponding 95% confidence intervals (CIs) for the PRs were calculated using the logarithmic method based on the observed numbers of cases and participants in the respective BMI categories. All statistical tests were two‐sided, and a *p* value < 0.05 was considered statistically significant.

## 3. Results

The general characteristics of the study sample are presented in Table 1. The results indicate that the majority of participants (63.9%) were aged 40–50 years, whereas 36.1% were aged 30–39.9 years.

**TABLE 1:** General characteristics of the study sample.

Variable	Classification	N (%)
Age (years)	30–39.9	83 (36.1)
	40–50	147 (63.9)

Educational level	High school	110 (47.8)
	≥ High school	120 (52.2)

Monthly income (JOD)	< 500	85 (37.0)
	≥ 500	145 (63.0)

Smoking	Yes	108 (47.0)
	No	122 (53.0)

Physical activity levels	Low (0–8)	49 (21.3)
	Moderate (9–17)	122 (53.0)
	High (18–28)	59 (25.7)

BMI‐C	Normal weight 18.5–24.9 kg/m^2^	80 (34.8)
	Overweight 25–29.9 kg/m^2^	60 (26.1)
	Obesity ≥ 30 kg/m^2^	90 (39.1)

Risk categories	Low < 10.0%	152 (66.1)
	Moderate (10–< 20%)	38 (16.5)
	High (≥ 20%)	40 (17.4)

*Note:* Values are presented as *N* (%).

Abbreviation: BMI‐C, body mass index classification.

This suggests a concentration of the sample in middle age, a period known for an increased risk of obesity and heart disease. In terms of educational level, the distribution was similar between those with less than a secondary education (47.8%) and those with a secondary education or higher (52.2%). However, the economic status showed some variation, with 63% of participants having a monthly income ≥ 500 Jordanian dinars. In contrast, 37% had an income of less than 500 Jordanian dinars.

From a behavioral perspective, 47% of the participants were current smokers, a relatively high percentage that may contribute to an increased risk of chronic diseases and heart disease. The majority of the sample had a moderate level of physical activity (53%), while 25.7% were highly active and 21.3% were low‐activity, indicating that approximately one‐fifth of the participants had insufficient physical activity, an established risk factor for obesity and CVD.

Regarding physical health indicators, the data showed a high prevalence of obesity at 39.1%, compared to 34.8% with a normal weight and 26.1% with being overweight. This indicates that obesity is a major health problem within the sample and may affect cardiovascular health and metabolism. According to the female ATP III Framingham point‐based algorithm, the estimated 10‐year hard CHD risk was low (< 10%) in 66.1% of participants, moderate (10–< 20%) in 16.5%, and high (≥ 20%) in 17.4%.

The distribution of biochemical and blood pressure parameters across BMI categories is presented in Table 2; increasing BMI was significantly associated with worsening cardiometabolic parameters. Obese participants exhibited significantly higher systolic and diastolic blood pressure, FBG, TC, LDL‐C, and TG compared to normal‐weight individuals (*p* < 0.001). Conversely, HDL‐C levels decreased significantly with increasing BMI. These findings highlight the strong association between obesity and adverse metabolic and cardiovascular risk profiles.

**TABLE 2:** Distribution of the mean values of serum lipid profiles, fasting blood glucose, and systolic and diastolic blood pressure according to body mass index categories.

	BMI‐C	Mean ± SD		BMI‐C	Mean difference ± STD error		*p*‐value
SBP	1	113.83	7.73	2–1	9.17	3.81	0.049
	2	123.00	8.06	3–2	15.73	3.11	0.000
	3	138.73	17.88	3–1	24.90	3.11	0.000

DBP	1	72.80	6.94	2–1	1.93	2.73	0.780
	2	74.73	6.65	3–2	12.81	2.23	0.00
	3	87.54	12.41	3–1	14.44	4.89	0.000

FBG	1	74.77	11.36	2–1	11.06	4.89	0.003
	2	91.70	24.81	3–2	27.99	4.99	0.024
	3	102.76	18.75	3–1	27.99	3.99	0.000

TC	1	172.35	35.09	2–1	24.55	9.86	0.048
	2	196.90	32.74	3–2	23.84	8.05	0.014
	3	220.74	40.74	3–1	48.39	8.05	0.000

LDL‐C	1	115.77	39.65	2–1	25.05	10.92	0.004
	2	140.82	42.790	3–2	50.69	8.95	0.000
	3	166.45	43.25	3–1	25.63	8.95	0.008

HDL‐C	1	53.02	7.92	2–1	5.75	1.76	0.006
	2	47.27	6.36	3–2	6.08	1.44	0.000
	3	41.19	6.59	3–1	11.83	1.44	0.000

TG	1	131.23	17.64	2–1	26.13	11.27	0.007
	2	157.24	30.86	3–2	29.83	9.20	0.000
	3	187.08	52.315	3–1	55.85	9.20	0.006

*Note:* Triglycerides (TG), results of one‐way ANOVA and Scheffe post hoc analysis: Category 1—normal BMI (*n* = 80), Category 2—overweight BMI (*n* = 60), Category 3—obese BMI (*n* = 90), STD, standard error.

Abbreviations: BMI‐C, body mass index categories; DBP, diastolic blood pressure; FBG, fasting blood glucose; HDL‐C, high‐density lipoprotein cholesterol; LDL‐C, low‐density lipoprotein cholesterol; SBP, systolic blood pressure; TC, total cholesterol.

Table 3 presents the prevalence and PRs of cardiometabolic abnormalities across BMI categories. Obese participants showed higher prevalences of abnormal lipid profiles, elevated FBG, and elevated blood pressure compared with normal‐weight participants. The highest PRs were observed for elevated diastolic blood pressure (PR2 = 9.19) and SBP (PR2 = 6.67).

**TABLE 3:** Prevalence and prevalence ratios of cardiometabolic abnormalities according to BMI categories.

BMI‐C/cardiometabolic abnormalities	Normal 18.5–24.9 kg/m^2^ (*N* = 80)	Overweight 25–< 30 kg/m^2^ (*N* = 60)	PR1 (95% CI)	Obese ≥ 30 kg/m^2^ (*N* = 90)	PR2 (95% CI)	*p*‐value
↑ TC	12 (15.0)	21 (35.0)	2.33 (1.25–4.36)	64 (71.1)	4.74 (2.77–8.12)	< 0.001
↑ LDL‐C	14 (17.5)	22 (36.7)	2.10 (1.17–3.74)	76 (84.4)	4.83 (2.97–7.83)	< 0.001
↓ HDL‐C	15 (18.8)	28 (46.7)	2.49 (1.46–4.23)	62 (68.9)	3.65 (2.28–5.92)	0.002
↑ TG	14 (17.5)	23 (38.3)	2.19 (1.23–3.89)	49 (54.4)	3.11 (1.86–5.19)	< 0.001
↑ FBG	9 (11.3)	12 (20.0)	1.78 (0.80–3.94)	45 (50.0)	4.44 (2.32–8.51)	< 0.001
↑ SBP	10 (12.5)	30 (50.0)	4.00 (2.12–7.53)	75 (83.3)	6.67 (3.71–11.99)	< 0.001
↑ DBP	6 (7.5)	12 (20.0)	2.67 (1.06–6.70)	62 (68.9)	9.19 (4.20–20.08)	< 0.001

*Note:* Values are presented as *n* (%). Normal BMI was used as the reference category (PR = 1.00). PR1 indicates the prevalence ratio for overweight versus normal BMI, and PR2 indicates the prevalence ratio for obesity versus normal BMI.

The association between demographic, behavioral, and obesity‐related variables with CHD risk categories is presented in Table 4. Age, smoking status, physical activity, and obesity indices were significantly associated with CHD risk categories (*p* < 0.001). A higher proportion of women aged 40–50 years were classified in the moderate and high‐risk groups compared to younger participants.

**TABLE 4:** Distribution of demographic, behavioral, and obesity‐related characteristics according to estimated 10‐year hard coronary heart disease risk categories.

Variables	Low < 10%	Moderate 10–< 20%	High ≥ 20%	Total	*p*‐value
*Age*					
30–39.9	76 (91.6)	2 (2.4)	5 (6.0)	83 (36.1)	0.000
40–50	76 (51.7)	36 (24.5)	35 (23.9)	147 (63.9)	

*Smoking*					
Yes	52 (48.2)	25 (23.1)	31 (28.7)	108 (47.0)	0.000
No	100 (82.0)	13 (10.6)	9 (7.4)	122 (53.0)	

*Physical activity levels*					
Low (0–8)	23 (47.0)	7 (16.3)	19 (36.7)	49 (21.3)	0.001
Moderate (9–17)	83 (68.0)	21 (17.2)	18 (14.8)	122 (53.0)	
High (18–28)	46 (78.0)	10 (16.9)	3 (5.1)	59 (25.7)	

*BMI-C*					
Normal weight 18.5–24.9 kg/m^2^	75 (93.7)	5 (6.3)	0 (0.0)	80 (34.8)	0.000
Overweight 25–< 30 kg/m^2^	42 (70.0)	11 (18.3)	7 (11.7)	60 (26.1)	
Obesity ≥ 30 kg/m^2^	35 (38.9)	22 (24.4)	33 (36.7)	90 (39.1)	

*WC*					
Low < 80 cm	75 (91.5)	3 (3.9)	0 (0.0)	78 (33.9)	0.000
Moderate 80–< 88 cm	42 (72.4)	12 (20.7)	4 (6.9)	58 (25.2)	
High ≥ 88 cm	35 (37.2)	23 (24.5)	36 (38.3)	94 (40.9)	

*WHtR*					
Low < 0.50	72 (94.7)	3 (4.0)	1 (1.3)	76 (33.1)	0.000
Moderate 0.50–< 0.55	47 (74.6)	11 (17.5)	5 (7.9)	63 (27.3)	
High ≥ 0.55	33 (36.2)	24 (26.4)	34 (37.4)	91 (39.6)	

*Note:* Values are presented as *n* (%). An estimated 10‐year hard CHD risk was calculated using the female‐specific ATP III Framingham point‐based algorithm and categorized as low (< 10%), moderate (10–< 20%), and high (≥ 20%).

Smoking and low physical activity were strongly associated with increased CHD risk. Notably, 36.7% of participants with low physical activity were classified as high risk, compared to only 5.1% among those with high activity levels.

Both general and central obesity indices (BMI, WC, and WHtR) were significantly associated with higher CHD risk. Participants with BMI ≥ 30 kg/m^2^ or elevated central obesity measures showed a markedly higher proportion of high‐risk classification compared to those with normal values.

## 4. Discussion

This study provides a comprehensive evaluation of the estimated 10‐year risk of CHD and its associated determinants among Jordanian women aged 30–50 years in northern Jordan. The findings revealed a high prevalence of general obesity (39.1%) and central obesity based on WC ≥ 88 cm (40.9%), both of which were associated with adverse cardiometabolic profiles and higher estimated CHD risk. Notably, 17.4% of participants were classified as being at high estimated risk according to the FRS. Collectively, these findings emphasize the potential contribution of age‐related changes and modifiable lifestyle factors, particularly smoking and physical inactivity, to cardiovascular risk among Jordanian women.

### 4.1. Demographic and Behavioral Risk Factors

Approximately 63.9% of the participants were between 40 and 50 years of age, representing the age range during which many women experience the menopausal transition. However, because menopausal status was not clinically assessed in the present study, the findings should be interpreted as representing women aged 30–50 years rather than a population classified according to menopausal status. Previous studies have identified midlife as a critical period for the development of obesity and metabolic syndrome due to hormonal changes, particularly declining estrogen levels and increased visceral fat accumulation [11]. Furthermore, age is an established independent determinant of CHD risk and constitutes one of the principal components of the FRS [19–22].

The high prevalence of smoking (47.0%) observed in the present study represents an important public health concern and a major modifiable risk factor for CVD. This finding is consistent with national reports indicating that Jordan has one of the highest smoking rates worldwide [23]. Smoking may exacerbate the adverse effects of central obesity by promoting oxidative stress, endothelial dysfunction, systemic inflammation, and reductions in HDL cholesterol, thereby accelerating the development of atherosclerosis [9]. More than one‐quarter of smokers in the present study were classified as being at high risk for CHD, highlighting the urgent need for targeted smoking cessation programs among Jordanian women. These findings are consistent with previous epidemiological studies demonstrating that smoking substantially increases cardiovascular morbidity and mortality through multiple pathophysiological mechanisms [23–29].

Physical activity was also associated with the estimated cardiovascular risk in the present study, with lower physical activity levels generally observed among women with higher estimated risk. However, because the adapted physical‐activity instrument was not subjected to formal criterion‐related or construct‐validity testing, these findings should be interpreted cautiously, consistent with previous evidence demonstrating that regular moderate‐to‐vigorous physical activity improves insulin sensitivity, body weight regulation, lipid metabolism, and overall cardiovascular health [2, 27, 30, 31]. Only 5.1% of women with high levels of physical activity were classified as being at high CHD risk, compared with 36.7% of those reporting low physical activity. These findings provide strong local evidence supporting the promotion of regular physical activity as a cornerstone of CVD prevention among Jordanian women.

The present findings also reinforce previous evidence indicating that central obesity is significantly associated with cardiometabolic and cardiovascular risk and may represent an important target for preventive interventions [5, 11].

### 4.2. Obesity as a Major Determinant of Cardiometabolic Dysfunction

The present study clearly demonstrates the detrimental impact of obesity on cardiometabolic health. A significant dose–response relationship was observed between BMI categories and all measured cardiometabolic parameters (Tables 2 and 3). Obese participants exhibited significantly higher systolic and diastolic blood pressure, FBG, TC, LDL cholesterol, and triglyceride levels, together with significantly lower HDL cholesterol concentrations. These findings are characteristic of metabolic syndrome and are consistent with previous international and regional studies conducted in Jordan and other Middle Eastern populations [1, 17, 23, 25–29].

Several biological mechanisms may explain these associations. Excess visceral adiposity promotes chronic low‐grade inflammation through increased secretion of proinflammatory adipokines, including tumor necrosis factor‐α (TNF‐α) and interleukin‐6 (IL‐6), while simultaneously contributing to insulin resistance, oxidative stress, endothelial dysfunction, and activation of the RAAS [11, 17, 32, 33]. Together, these mechanisms promote dyslipidemia, hyperglycemia, hypertension, and ultimately accelerate the development of atherosclerosis. The markedly elevated PRs observed among obese participants further support the strong relationship between increasing adiposity and cardiometabolic abnormalities.

These findings are consistent with previous studies from Jordan and neighboring Middle Eastern countries demonstrating that WC and WHtR are strong predictors of metabolic syndrome and cardiovascular risk [25–30].

### 4.3. Obesity and Estimated 10‐Year CHD Risk

High estimated CHD risk was more common among women with general and central obesity. Specifically, 36.7% of women with obesity (BMI ≥ 30 kg/m^2^), 38.3% of those with WC ≥ 88 cm, and 37.4% of those with WHtR ≥ 0.55 were classified as having high estimated CHD risk. These findings indicate a strong association between adiposity and estimated 10‐year CHD risk; however, the cross‐sectional design and absence of multivariable modeling preclude conclusions regarding causality or independent effects.

This observation supports accumulating evidence that central fat distribution is a more clinically relevant predictor of cardiometabolic risk than overall adiposity, particularly among middle‐aged women [32, 33]. Nevertheless, because the FRS has not been specifically validated in Jordanian women, the estimated absolute CHD risk reported in this study should be interpreted cautiously.

### 4.4. Study Limitations

This study has several limitations. First, its cross‐sectional design precludes the establishment of causal relationships between obesity and CHD risk. Second, participants were recruited from a single semiurban community in northern Jordan, which may limit the generalizability of the findings to the broader Jordanian population. Third, approximately 63.9% of participants were aged 40–50 years, representing an age range in which the menopausal transition may occur. However, menopausal status was not clinically assessed in the present study; therefore, the findings should be interpreted as representing women within this age group rather than a population classified according to menopausal status.

Fourth, although the FRS is widely used internationally, it has not been specifically validated in Jordanian women; therefore, the estimated absolute cardiovascular risk should be interpreted with caution. Finally, multivariable regression analyses were not performed, limiting the ability to determine the independent contributions of obesity, smoking, physical activity, and other cardiometabolic risk factors to estimated CHD risk.

## 5. Conclusion

Jordanian women aged 30–50 years exhibited a substantial burden of both general and central obesity, accompanied by an increased estimated risk of CHD. These findings support the implementation of routine cardiovascular risk assessment and comprehensive lifestyle interventions focusing on weight management, smoking cessation, and regular physical activity. Although the FRS provides a practical framework for cardiovascular risk stratification, its estimates should be interpreted cautiously because the model has not been specifically validated in Jordanian women. Future prospective, multicenter studies incorporating population‐specific cardiovascular risk prediction models and multivariable analyses are warranted to validate and extend the present findings.

## Funding

No funding was received for this manuscript.

## Conflicts of Interest

The authors declare no conflicts of interest.

## Data Availability

The data that support the findings of this study are available on request from the corresponding author. The data are not publicly available due to privacy or ethical restrictions.
